# Why Cognitive–Cognitive Dual-Task Testing Assessment Should Be Implemented in Studies on Multiple Sclerosis and in Regular Clinical Practice

**DOI:** 10.3389/fneur.2020.00905

**Published:** 2020-08-28

**Authors:** Christian Beste, Tjalf Ziemssen

**Affiliations:** ^1^Cognitive Neurophysiology, Department of Child and Adolescent Psychiatry, Faculty of Medicine, TU Dresden, Dresden, Germany; ^2^Department of Neurology, Faculty of Medicine, Centre of Clinical Neuroscience, MS Centre Dresden, TU Dresden, Dresden, Germany

**Keywords:** multiple sclerosis, cognition, dual tasking, e-health, neuropsychology

## Abstract

Cognitive impairment is prevalent and disabling in multiple sclerosis (MS) and is severely impacting quality of life (QoL). Aside its routine assessment in clinical care, it should more often be implemented as endpoint/outcome measure in clinical trials. However, a fundamental aspect—often neglected in clinical practice and clinical trials—is the assessment of multi-tasking and dual-tasking abilities. In this perspective article, we outline why, given the nature of MS, particularly the assessment of “cognitive–cognitive dual-tasking” is relevant in MS. We delineate how knowledge from basic cognitive science can inform the assessment of this important cognitive impairment in MS. Finally, we outline how the assessment of “cognitive–cognitive dual-tasking” can be implemented in computer-based screening tools (e-health devices) that can be used not only in clinical diagnostics but also in clinical trials.

## Introduction

Cognitive impairment is prevalent at all phases and all subtypes of multiple sclerosis (MS). It remains one of the major causes of neurological disability in young and middle-aged adults suffering from the disease (1). The severity and the type of cognitive impairment vary considerably among individuals and can be observed both in early and in later stages. The usual neurological examination fails to detect emerging cognitive deficits; self-reported cognitive complaints by the patients can be confounded by other subjective symptoms (2), so the assessment of cognitive functions should become a cornerstone in routine clinical care of MS patients and is also increasingly considered as an important endpoint in clinical trials (3). Especially with regard to the inclusion of cognitive tests in clinical trials, it is essential that the tests are reliable and quickly feasible. Based on these grounds, especially the symbol digit modalities tests (SDMT) has been included in recent clinical trials. This is also reasonable because the SDMT has been considered to reflect a reliable and relevant cognitive screening instrument in MS (4, 5). The SDMT mainly measures perceptual and attentional speed. Although these are central dysfunctions in MS and, of course, relevant for the patients, MS patients also complain about difficulties when being confronted with “multi-tasking” situations (e.g., in job occupation) (1). Although deficits in these abilities are frequently reported by MS patients, they are not routinely examined, which is a fundamental shortcoming (6). Often there is a strong discrepancy in a patient's statements about difficulties occurring in daily life and the pattern of the neuropsychological profile as revealed by routinely applied neuropsychological test (batteries) in MS. This is likely the case because current testings (including the SDMT) fall short of examining relevant cognitive dual- or multi-tasking abilities. Distinctions have been made between different forms of multi-tasking (6), and purely cognitive dual-tasking situations have been distinguished from situations in which cognitive and motor demands are imposed in parallel—that is, a distinction between cognitive–cognitive and cognitive–motor dual-tasking situation has been made. The latter (cognitive–motor dual-tasking situations) has already been subject to intense research in MS, and several studies and review articles have been published on walking and postural balance (7–10). However, these sorts of dual-tasking assessment require specialized hard and software packages and cumbersome presentation devices. The clinical usage and the dissemination of “dual-tasking assessments” are strongly facilitated, and their acceptance is increased if a test is short and can, ideally, be delivered flexibly (i.e., without specific software requirements and hardware devices in various settings). This is the case for cognitive–cognitive dual-tasking assessments as outlined below.

## Cognitive–Cognitive Dual Tasking Assessment in the Context of Ms

For these matters, especially the assessment of executive functions is central because executive functions predict performance in many daily life relevant areas as [e.g., job occupation (11)]. Especially in MS, this is central since this disease mostly affects people between 20 and 50 years of age. However, executive functions cover a wide range of cognitive processes. Therefore, the exact examination of executive functions often requires various tests so that the examination is time-consuming and rarely feasible when testing novel pharmacological compounds in clinical study settings. A further problem is that many everyday situations do not only claim a circumscribed executive function but represent a mixture of different processes. For this reason, cognitive testing using common neuropsychological tests often falls short (1, 6). Most day-to-day requirements demand several aspects of executive functions simultaneously or in rapid succession.

**Figure 1:**
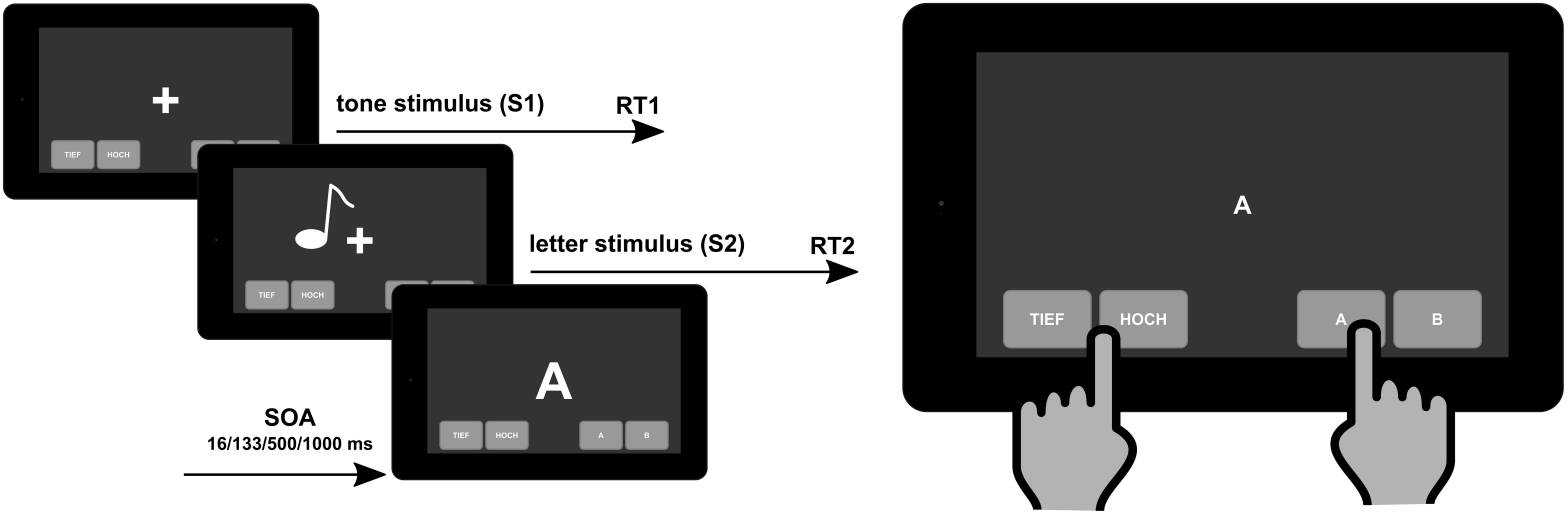
Schematic illustration of the psychological refractory period task to measure “cognitive–cognitive” dual tasking (left) that can be implemented on a mobile e-health device (right). SOA, stimulus onset asynchrony.

Dual-tasking, and its assessment, captures the interaction of different executive functions and therefore comes closer to requirements in everyday life. As such, the assessment of dual-tasking functions is important and has an ecological validity for the assessment of cognitive dysfunctions associated with MS (12). Over the last decades, especially research in cognitive and experimental psychology has uncovered the cognitive mechanisms involved in dual- and multi-tasking (13). Importantly, this research has developed rigorous methods (i.e., tests) to assess these functions. One of the most established tests is the psychological refractory period (PRP) task (14) and derivatives of it, like a stop-change task (15). Briefly, people are required to execute two responses in close succession to two different streams of stimuli (e.g., visual and auditory stimuli) (see Figure 1, left side, for illustration).

When these responses are demanded in close succession, response selection capacities become overstrained and response selection processes are slowed down (13, 16, 17). Several lines of evidence suggest that these capacities depend on brain structures in the frontal, fronto-central, and parietal regions and are thus organized as long-distance functional neuroanatomical networks (18–27). This is of particular relevance for MS because MS can be seen as a white matter disconnection syndrome (28). Consequently, it has been shown that the ability to select appropriate responses in close succession is predicted by concentrations of serum neurofilament light chain (sNfl) (29). This is of high relevance because there are strong links between the sNfl and the integrity of the white matter structure (30–34), particularly in MS (35, 36). It has been shown (37) that MS patients performed considerably worse than healthy control participants and that the deficits shown by the patients are very likely not due to simple motor deficits.

Aside these neuroanatomical and neurobiological considerations suggesting that the assessment of dual-tasking is relevant in MS, it is important to note that these abilities show little to no susceptibility to learning effects (38). Only after an extensive several-hours training will slight changes in dual-tasking abilities have been documented (39). This is important because the cognitive function (construct) being tested remains reliably testable across different testings (i.e., longitudinally). This contrasts with other tests routinely used to assess cognitive functions in MS, like the PASAT, where strong learning effects are evident and patients report that they do not attend to the task because they already know what is being presented one after another in this task (40). Thus, the assessment of dual-tasking in MS is desirable because the same cognitive function is always tested and not the mixing of learning skills/effects and the cognitive function to be measured. This is furthermore the case because dual-tasking tests (like the PRP) require the responses to be simple visual digits/letters and tones, making it possible to create parallel versions of the task easily and quickly without changing the task difficulty or other characteristics of the test. Data examining the PRP in MS have shown that variations in motor speed (e.g., due to MS-related motor disturbances) do not represent a confound in this task because mostly the accuracy to respond seems to be modulated in MS (37). Moreover, a PRP task can also be applied using voice responses (41). As mentioned, the mechanisms underlying dual-tasking have been subject to intense research for many decades. This has led to an in-depth knowledge of the cognitive subprocesses underlying dual-tasking abilities, with the result that performance in dual-tasking can be described with well-established mathematical models (13). Aside the fact that this underlines the high reliability and validity of the testing procedure, it ensures that the tested cognitive processes are consistent and quantitative. Due to its mathematical modelability, it can be described very clearly under which specific test constellations (test difficulties) differences between persons can be reliably measured. This is important given the (partially) progressive nature of MS and the necessity to be able to track disease progression also at a cognitive level. The strong conceptual rationale has driven knowledge gain on the cognitive processes being important during dual- and multitasking as well as a task design which ensures that “adaptive” testing is possible and ensures to record longitudinal data with one test without having to change the evaluation instrument. On the same grounds, dual-tasking (and especially the PRP) is reliable and quick to apply, with a high degree of standardization. We have developed a tablet-based solution which can be applied to the patient without extensive explanations. This has two important consequences: first, the test is easy to apply, without intense training of nurses in the clinical real world as well as in study settings and, second and more important, these features of dual-tasking assessment using the PRP enable an assessment using digital health devices which could be applied in MS centers or by the patient himself. This ensures that a dual-tasking assessment using the PRP (and related tasks) is quickly scalable to high case numbers in the context of clinical study situations. In addition, this clinically very relevant test could be transferred to everyday clinical practice to monitor cognitive function longitudinally. The validity of such cognitive tests can be related to two general concepts. The first is construct or concept validity which is quite clear about the dual-task challenge. The other principle includes quantitative interpretability (42). The FDA guidance does not see the treatment benefit as a purely statistical issue but, rather, that it is important to also be able to interpret the observed treatment effect as clinically meaningful. The identification of a score difference can be interpreted as a treatment benefit (i.e., clinically meaningful). Up to now, the SDMT as single, mental processing speed test has been used in clinical studies so far, and it will be important to be able to replicate the results in the domain of executive function which is often defective in MS patients and has the above-mentioned advantages of testing. Data from cognitive tests such as the dual-task test with both statistically and clinically meaningful approaches are needed.

Importantly, the nature and the structure of the PRP dual-task assessment makes it possible to implement this neuropsychological tool in e-health devices [i.e., tablet-based applications that can be on the “bedside” and in routine clinical care in outpatient units (see Figure 1, right side)]. The e-health diagnostic tools are helpful instruments to close the supply shortfall in the healthcare system and to improve the care of chronically ill patients because they can present the course of the illness more comprehensively and more accurately than only through standard clinical visits. The MS patients are a suitable group of e-health users (43). Using digital tools, data collection does not increase so much the burden on providers or generate a significant incremental cost, so the proliferation of computerized neuropsychological assessment devices for screening and monitoring cognitive impairment is increasing exponentially (44). In our approach, the digital dual-task assessment tool is implemented in our Multiple Sclerosis Documentation Software MSDS3D and the linked Integrated Care Portal Multiple Sclerosis (IBMS) which contains clinical pathways in a manner which is comprehensible for the patients (45). This is in line with our overall strategy toward personalized MS management such that, in addition to advanced immunological, genetic, and MRI profiling of the individual patient, the clinical profiling of MS patients' inclusive cognition needs to be widely implemented in clinical practice using digital approaches (46).

## Conclusion

We hope that the self-explanatory reviewed cognitive–cognitive dual-task test will lower the threshold for regular cognitive testing. This has to be proven in future clinical studies. The unsupervised assessment of dual-task function is time-efficient and comes with an advantage that scores could be automatically calculated and sent to the treating neurologist immediately, so regular digital dual-task testing as cognitive monitoring in MS patients will be possible. Ultimately, performing a dual-task test will provide clinicians with an indication of the cognitive performance of patients with MS without the need of a test leader. Follow-up measurement will be easier to implement and could lead to the timely identification of cognitive decline in patients with MS and subsequently allow for adequate counseling. Focusing at clinical studies, it will be easier to investigate cognitive function as a primary outcome.

## Data Availability Statement

The original contributions presented in the study are included in the article/supplementary material, further inquiries can be directed to the corresponding author/s.

## Author Contributions

All authors conceived the theoretical outline of the article, written, and approved the manuscript.

## Conflict of Interest

The authors declare that the research was conducted in the absence of any commercial or financial relationships that could be construed as a potential conflict of interest.
